# Harnessing the Power of AI: A Comprehensive Review of Its Impact and Challenges in Nursing Science and Healthcare

**DOI:** 10.7759/cureus.49252

**Published:** 2023-11-22

**Authors:** Seema Yelne, Minakshi Chaudhary, Karishma Dod, Akhtaribano Sayyad, Ranjana Sharma

**Affiliations:** 1 Nursing, Shalinitai Meghe College of Nursing, Datta Meghe Institute of Higher Education and Research, Wardha, IND

**Keywords:** interdisciplinary collaboration, patient outcomes, ethical challenges, nursing science, healthcare, artificial intelligence (ai)

## Abstract

This comprehensive review delves into the impact and challenges of Artificial Intelligence (AI) in nursing science and healthcare. AI has already demonstrated its transformative potential in these fields, with applications spanning from personalized care and diagnostic accuracy to predictive analytics and telemedicine. However, the integration of AI has its complexities, including concerns related to data privacy, ethical considerations, and biases in algorithms and datasets. The future of healthcare appears promising, with AI poised to advance diagnostics, treatment, and healthcare practices. Nevertheless, it is crucial to remember that AI should complement, not replace, healthcare professionals, preserving the essential human element of care. To maximize AI's potential in healthcare, interdisciplinary collaboration, ethical guidelines, and the protection of patient rights are essential. This review concludes with a call to action, emphasizing the need for ongoing research and collective efforts to ensure that AI contributes to improved healthcare outcomes while upholding the highest standards of ethics and patient-centered care.

## Introduction and background

Artificial Intelligence (AI) in healthcare refers to using advanced technologies and algorithms to replicate human cognition and decision-making processes in the analysis, interpretation, and management of medical data [1]. In nursing science and healthcare, AI encompasses various applications, ranging from predictive analytics and data-driven insights to virtual nursing assistants and robotic surgical systems. By leveraging AI, healthcare professionals can augment their capabilities and improve the quality, efficiency, and precision of patient care delivery [1].

Integrating AI in nursing science and healthcare has emerged as a transformative force, revolutionizing how healthcare services are delivered, managed, and optimized. AI technologies have the potential to enhance clinical decision-making, streamline administrative processes, and facilitate the development of personalized treatment plans tailored to individual patient needs. Moreover, AI-driven solutions offer a promising avenue for addressing the challenges of resource constraints, increasing patient loads, and the growing complexity of healthcare data, thereby contributing to improved patient outcomes and a more sustainable healthcare ecosystem [2].

The primary purpose of this comprehensive review is to critically examine the multifaceted impact of AI on nursing science and healthcare. By analyzing the current landscape of AI integration, exploring its implications for patient care, and addressing the challenges and ethical considerations associated with its implementation, this review aims to provide a nuanced understanding of the opportunities and limitations presented by AI in the healthcare domain. Additionally, this review seeks to offer insights into the prospects and opportunities for harnessing AI's potential in nursing science and healthcare while providing actionable recommendations for optimizing its integration into clinical practice and healthcare management.

## Review

The current landscape of AI integration in nursing science and healthcare

Overview of AI Applications in Healthcare Settings

Medical Imaging: In medical imaging, AI is pivotal in improving diagnostic accuracy and efficiency. By analyzing x-rays, MRIs, and CT scans, AI algorithms can detect subtle anomalies, making early disease detection possible. For instance, in the context of mammography, AI can help identify breast cancer at its earliest stages, greatly enhancing the chances of successful treatment. AI-powered medical imaging not only speeds up interpreting these images but also assists radiologists and other healthcare professionals in providing more accurate diagnoses [3].

Clinical decision support: AI-driven clinical decision support systems are designed to work alongside healthcare professionals, offering real-time guidance and recommendations. These systems help in diagnosing diseases, selecting appropriate treatment options, and issuing alerts about potential issues. For instance, an AI system can analyze a patient's medical history, symptoms, and test results to provide doctors with evidence-based treatment options, aiding decision-making and improving patient care. These systems also help reduce the likelihood of medical errors [4].

Healthcare analytics: AI-driven healthcare analytics is a game-changer for healthcare organizations. With the vast amounts of data generated in healthcare settings, AI can efficiently process and analyze this data to derive valuable insights. This analysis includes predicting patient outcomes, optimizing resource allocation, and improving patient care. For example, AI can assist hospitals in forecasting patient admission rates, allowing for better staff scheduling and resource allocation, ultimately enhancing the quality of care while controlling costs [5].

Natural language processing (NLP): NLP algorithms are essential for unlocking valuable information hidden within unstructured clinical notes and medical records. These algorithms can extract relevant data from text, such as physician's notes or patient records, and convert it into structured and actionable information. NLP aids in efficient record-keeping and data retrieval, making it easier for healthcare professionals to access and utilize patient data. Additionally, it is crucial to enable electronic health records to be more beneficial for clinical decision support, research, and data analysis [1].

Robotics: Integrating AI in robotics has brought significant advancements in surgical precision and minimally invasive procedures. Surgical robots, guided by AI, can perform complex procedures with exceptional accuracy. They provide surgeons with enhanced dexterity, control, and magnified 3D views, allowing for greater precision and smaller incisions. This reduces patient trauma, shorter recovery times, and improves surgical outcomes. Robotics in surgery exemplifies the synergy between AI and healthcare, with the potential to redefine the future of surgical practices and patient care [6].

AI-Powered Technologies in Nursing Practice

Virtual nursing assistants: Virtual nursing assistants powered by AI are increasingly being integrated into healthcare settings to support nurses. These AI-driven chatbots and virtual assistants assist nurses in various tasks, such as managing patient records, accessing up-to-date medical information, and answering routine questions. They enhance workflow efficiency by quickly retrieving patient data, enabling nurses to focus more on direct patient care. Moreover, virtual nursing assistants can help with appointment scheduling, medication reminders, and post-discharge follow-ups, improving patient engagement and satisfaction [1].

Medication management: AI systems are vital in ensuring accurate and timely medication administration, a critical aspect of patient care. These systems help nurses by providing automated medication reminders and dose calculations, reducing the risk of medication errors. AI can also cross-reference patient records and prescriptions, alerting nurses to potential drug interactions or allergies. By streamlining the medication management process, nurses can dedicate more time to patient care, and patients benefit from safer and more effective treatments [7].

Fall prediction: Fall prevention is a crucial aspect of patient safety, particularly for elderly or at-risk patients. AI can analyze patient data, such as gait patterns, balance, and medical history, to predict the risk of falls. This predictive capability enables nurses to take proactive measures to prevent falls, such as adjusting patient care plans, providing mobility aids, or instituting regular check-ins. By leveraging AI for fall prediction, nurses can significantly enhance patient safety and reduce the incidence of fall-related injuries [8].

Patient monitoring: AI-driven patient monitoring has revolutionized the way healthcare professionals keep track of patient well-being. Wearable devices and remote monitoring systems equipped with AI continuously collect and analyze vital signs, such as heart rate, blood pressure, and oxygen levels. When irregularities or concerning trends are detected, these systems can automatically alert nurses and healthcare providers, allowing for timely interventions. This real-time monitoring capability is especially beneficial for patients with chronic conditions or those recovering from surgery, as it enables earlier intervention and reduces the need for constant in-person oversight. Nurses can allocate their time more efficiently, focusing on patients who require immediate attention, thus improving the quality of care and patient outcomes [9].

AI-Driven Advancements in Patient Care and Treatment

Personalized treatment plans: AI-driven personalized treatment plans are at the forefront of the shift towards precision medicine. AI algorithms analyze a patient's comprehensive health data, including genetics, medical history, lifestyle, and real-time health metrics, to design tailored treatment strategies. This personalized approach allows healthcare providers to deliver treatments optimally suited to individuals' unique needs and characteristics. For instance, AI can recommend specific medications, dosages, and interventions based on the patient's genetic predispositions, minimizing adverse effects and enhancing treatment effectiveness. By personalizing treatment plans, healthcare professionals can provide more targeted and efficient care, ultimately improving patient outcomes and satisfaction [10].

Drug discovery: AI has revolutionized the drug discovery process by expediting the identification of novel drug candidates. AI-driven simulations enable the prediction of molecular interactions and the assessment of potential drug efficacy, significantly accelerating the research and development of new medications. This is particularly critical in addressing diseases for which treatment options are limited. AI systems can analyze vast datasets to identify promising compounds for further investigation, ultimately reducing the time and resources required to bring new drugs to the market. AI-powered drug discovery not only offers hope for faster and more cost-effective treatments but also opens up possibilities for personalized medicine tailored to an individual's genetic profile [11].

Remote healthcare: AI-driven telemedicine and remote healthcare services have become increasingly vital, especially in remote or underserved areas. Telemedicine platforms enable patients to access healthcare services from the comfort of their homes or local clinics, often with AI-enhanced diagnostic tools and virtual consultations. This could reduce barriers to healthcare access, such as geographic distance or limited healthcare infrastructure. AI plays a role in remote patient monitoring, wearable devices, and diagnostic tools, allowing healthcare professionals to remotely track patient vital signs and provide timely interventions when necessary. In this way, AI promotes healthcare equity by expanding access to quality care, improving patient management, and reducing the burden on healthcare facilities [12].

Early disease detection: AI algorithms excel in early disease detection by scrutinizing patient data for subtle changes that might indicate the onset of conditions like sepsis or chronic diseases. Identifying early warning signs allows healthcare professionals to intervene promptly, potentially preventing disease progression and reducing patient morbidity and mortality. For instance, AI can continuously monitor vital signs and laboratory results, automatically flagging anomalies or trends that could indicate a deteriorating patient condition. This early detection capability is precious in critical care settings, where timely interventions can be lifesaving. AI-driven early disease detection complements clinical judgment, offering a valuable safety net for healthcare providers and improved patient outcomes [13].

Examples of Successful AI Implementation in Nursing and Healthcare

IBM Watson for oncology: IBM Watson for Oncology is a pioneering AI system that offers invaluable support to oncologists in making critical treatment decisions. By analyzing an extensive database of medical literature, clinical trial data, and patient records, Watson for Oncology provides oncologists with personalized treatment recommendations for cancer patients. This AI system considers factors such as the patient's medical history, genetics, and the latest medical research, enabling oncologists to select the most suitable and evidence-based treatment options. The system does not replace oncologists but is an invaluable tool in augmenting their expertise, ultimately improving the quality of cancer care and increasing the likelihood of positive patient outcomes [14].

Google Health's DeepMind: DeepMind, a subsidiary of Google Health, has significantly contributed to the healthcare sector with its AI capabilities. In particular, DeepMind's AI algorithms have been utilized for predicting patient deterioration. By analyzing patient data, such as vital signs, laboratory results, and historical records, AI models can identify patterns and changes that might indicate patient deterioration. This early warning system allows clinicians to intervene proactively, potentially preventing adverse events and improving patient safety. DeepMind's Streams app complements these efforts by providing clinicians with an efficient platform to manage and prioritize patient data, ensuring that healthcare providers are well-informed and equipped to make timely decisions [15].

IDx-DR: IDx-DR is a groundbreaking AI-based system that received FDA approval for autonomous detection of diabetic retinopathy, a common complication of diabetes that can lead to vision loss if not promptly diagnosed and treated. This AI system analyses retinal images and, with a high degree of accuracy, identifies the presence of diabetic retinopathy. By automating the diagnostic process, IDx-DR addresses the shortage of eye care specialists in many regions and facilitates early detection and intervention, potentially saving patients from vision impairment. This approval underscores the potential of AI in automating routine diagnostic tasks, enhancing the efficiency of healthcare services, and expanding access to essential medical screenings [16].

PathAI: PathAI is a prime example of AI's transformative impact on pathology. By employing AI algorithms, PathAI supports pathologists in diagnosing diseases from histopathology slides with exceptional accuracy. These algorithms can detect subtle and complex patterns in tissue samples, aiding pathologists in making more precise and timely diagnoses. This not only enhances diagnostic accuracy but also streamlines the pathology workflow, reducing the time required for diagnosis and enabling faster treatment decisions. PathAI represents the successful synergy of human expertise and AI capabilities, promising to revolutionize pathology and improve patient care by improving diagnostic accuracy, particularly in complex and time-sensitive cases [17].

Impact of AI in nursing science and healthcare

Improved Patient Outcomes and Personalized Care

Enhanced decision support: AI has ushered in a new era of healthcare decision support by providing healthcare professionals access to a wealth of patient data and knowledge. This vast information repository encompasses medical literature, historical patient records, and real-time data, enabling more informed and evidence-based decision-making. With AI's assistance, healthcare professionals can analyze and interpret complex datasets rapidly and accurately. This leads to more precise diagnosis and treatment selection, ultimately improving patient outcomes. AI helps doctors and clinicians stay updated on the latest research and best practices, enhancing the quality of care delivered to patients [1].

Personalized treatment plans: AI's ability to analyze patient-specific information, including genetic data, medical history, and real-time health metrics, facilitates the creation of personalized treatment plans. By tailoring treatments to individual patient characteristics, such as genetic predispositions and lifestyle factors, AI enhances the likelihood of treatment success. This personalized approach minimizes adverse effects and increases treatment effectiveness as patients receive interventions better aligned with their needs. By integrating AI for personalized treatment plans, healthcare professionals can optimize patient care, improving health outcomes and patient satisfaction [18].

Early detection and intervention: AI is an indispensable tool for early disease detection and timely intervention. AI algorithms are adept at continuously monitoring patient data, such as vital signs and laboratory results, and identifying subtle changes that could signify deteriorating health conditions. This capability empowers healthcare professionals to take prompt action when needed, potentially saving lives. For instance, in the case of sepsis, AI can recognize early signs of the condition, alerting medical teams to intervene before it escalates. By leveraging AI for early detection and intervention, healthcare providers can improve patient safety, reduce the severity of illnesses, and enhance overall healthcare quality [19].

Remote patient monitoring: AI-powered remote patient monitoring has transformed how patients receive care, particularly those with chronic conditions or those recovering from surgery. With the aid of wearable devices and remote monitoring systems, patients can be closely tracked in the comfort of their own homes. AI algorithms continuously analyze data, including vital signs and patient-reported information, to identify potential issues or deviations from the norm. Healthcare professionals receive real-time alerts when necessary, allowing them to intervene remotely and offer timely guidance or care adjustments. This approach not only enhances patient comfort but also reduces the risk of hospital-acquired infections and unnecessary hospitalizations. AI-driven remote patient monitoring promotes patient autonomy, safety, and adherence to treatment plans, ultimately improving healthcare outcomes [20].

Enhanced Efficiency and Accuracy in Diagnosis and Treatment

Reduced diagnostic errors: AI systems in medical imaging have the potential to reduce diagnostic errors significantly. These AI algorithms, trained on extensive datasets and equipped with pattern recognition capabilities, can provide more accurate and consistent results than human radiologists in some instances. By detecting subtle anomalies and abnormalities that the human eye might miss, AI enhances the precision and reliability of diagnostic processes. This, in turn, reduces the likelihood of misdiagnosis and ensures that patients receive appropriate and timely treatment, ultimately improving patient safety and healthcare quality [21].

Streamlined workflow: AI-driven automation and support tools are instrumental in streamlining the healthcare workflow. By automating administrative tasks, such as data entry and appointment scheduling, AI reduces the administrative burden on healthcare professionals. These efficiency gains allow healthcare providers to allocate more time and attention to direct patient care, fostering a more patient-centered approach. The result is a smoother and more efficient healthcare system that enhances the quality of care and the overall patient experience, all while reducing the risk of human errors in administrative processes [22].

Speedier drug discovery: AI has brought about a revolution in drug discovery by significantly accelerating the drug development process. AI-driven algorithms can swiftly analyze vast datasets, simulating molecular interactions and identifying potential drug candidates. These speed up the early stages of drug development and reduce the time and resources required to bring new medications to market. The implications of this accelerated drug discovery process are profound, promising faster access to novel treatments for various diseases and conditions. AI in drug discovery can improve patient access to lifesaving and life-enhancing drugs while reducing the costs and time associated with research and development [23].

Predictive analytics: AI's predictive analytics capabilities can transform hospital operations. By analyzing historical and real-time data, AI can forecast patient admission rates, enabling hospitals to allocate resources effectively, maintain optimal staffing levels, and reduce patient wait times. This proactive approach ensures that hospitals are well-prepared to handle patient surges, resulting in improved patient satisfaction and efficient resource allocation. Predictive analytics powered by AI optimize healthcare operations and resource management, contributing to the overall quality of patient care and healthcare service delivery [1].

AI-Driven Advancements in Medical Research and Data Analysis

Data processing: AI's ability to swiftly process large datasets is a game-changer in medical research. The sheer volume and complexity of healthcare data can be overwhelming for human researchers. AI algorithms excel at identifying trends, patterns, and correlations that might remain hidden from the human eye. By rapidly analyzing and synthesizing data from diverse sources, AI accelerates the pace of medical research. It assists researchers in identifying potential breakthroughs, novel disease markers, or treatment strategies more efficiently. This not only saves time but also opens up new avenues for scientific exploration, ultimately benefiting patient care and public health [24].

Drug repurposing: AI is vital in identifying existing drugs that can be repurposed for new medical treatments. This can significantly reduce the time and costs associated with drug development. AI-driven algorithms analyze vast datasets of drug properties, medical literature, and patient outcomes to identify candidate drugs that may have overlooked potential. By repurposing existing medications for new uses, AI supports faster innovation in healthcare, offering patients access to treatments that have already undergone safety testing and approval. Drug repurposing demonstrates AI's capacity to enhance healthcare efficiency and cost-effectiveness, all while expanding the range of available treatment options [23].

Genomic medicine: AI's analysis of vast genomic datasets is instrumental in the emerging field of genomic medicine. AI-driven genomics can identify genetic predispositions to diseases and conditions, providing valuable insights for personalized medicine. By assessing an individual's genetic makeup, AI enables healthcare professionals to design more targeted and effective treatment plans. This approach considers an individual's genetic variations, allowing for treatments better aligned with their unique genetic profile. Genomic medicine holds the promise of tailoring healthcare to the individual, optimizing treatment outcomes, and reducing the risk of adverse effects [25].

Clinical trial optimization: AI contributes to the optimization of clinical trials, a critical step in the development of new medical treatments. AI can aid in designing more efficient and diverse clinical trials, potentially accelerating the development of new therapies. By analyzing vast patient data, AI can identify suitable candidates, ensuring that clinical trials include a broader and more representative range of participants. This not only expedites the research process but also increases the likelihood of discovering effective treatments for a broader spectrum of patients. AI-driven clinical trial optimization advances healthcare research and the development of new therapies, ultimately benefiting patient populations worldwide [26].

The Role of AI in Predictive Modelling and Preventive Care

Risk prediction: AI's capacity to assess patient data and predict individuals at higher risk for specific conditions is a significant advancement in healthcare. By analyzing a combination of health records, lifestyle data, and genetics, AI can identify early warning signs and risk factors. This information empowers healthcare professionals to intervene proactively, offering preventive care and early interventions to those at higher risk. For instance, AI can predict individuals at risk of developing diabetes or cardiovascular diseases and recommend lifestyle modifications or regular screenings. This risk prediction capability enhances patient outcomes by enabling timely interventions and reducing the disease burden [27].

Chronic disease management: AI plays a pivotal role in the continuous monitoring and management of chronic diseases, improving patient adherence to treatment plans and minimizing disease exacerbations. For patients with diabetes, asthma, or hypertension, AI-powered systems provide real-time data tracking and reminders for medications, lifestyle adjustments, and physician visits. These systems support patients in adhering to their prescribed treatments, reducing the risk of disease progression or complications. AI-driven chronic disease management contributes to better disease control and improved quality of life for patients with chronic conditions [28].

Population health management: AI offers valuable support to healthcare organizations in population health management. By analyzing patient data on a broader scale, AI can identify trends, risk factors, and health disparities within patient populations. Healthcare organizations can then use this information to design more proactive and preventive approaches. For example, AI can help identify regions with a high prevalence of certain diseases and tailor public health interventions accordingly. This population-based approach enhances healthcare equity, targets resources effectively, and contributes to improved overall health outcomes [1].

Epidemic surveillance: AI's ability to analyze data from various sources makes it a powerful tool for epidemic surveillance. AI algorithms can swiftly detect early signs of disease outbreaks by monitoring indicators such as symptom reports, hospital admissions, and social media activity. This early detection capability empowers public health agencies to respond rapidly to potential epidemics. By implementing timely interventions and control measures, they can limit the spread of diseases and protect public health. AI-driven epidemic surveillance not only helps mitigate the impact of outbreaks but also improves public health preparedness and response [29].

Challenges and ethical implications of AI implementation in healthcare

Data Privacy and Security Concerns

Patient data protection: The utilization of AI in healthcare heavily depends on access to patient data, including medical records, diagnostic images, and genetic information. While AI can potentially improve patient care and outcomes, it also raises significant concerns about data privacy. Safeguarding sensitive health information is crucial to maintaining patient trust and complying with healthcare regulations like HIPAA (Health Insurance Portability and Accountability Act). Robust security measures, such as encryption, access controls, and auditing, are essential to protect patient data from unauthorized access or breaches. Ethical data handling and adherence to stringent privacy regulations are fundamental to ensure that AI-driven healthcare remains safe and respectful of patient privacy [1].

Data breaches: The interconnected nature of healthcare systems, along with the growing reliance on electronic health records, makes the healthcare sector particularly vulnerable to cyberattacks and data breaches. Data breaches can compromise patient privacy and safety, as well as erode public trust in healthcare providers. The consequences of such breaches can be severe, leading to identity theft, fraudulent medical claims, and even incorrect medical treatments. Robust cybersecurity measures, regular system updates, and staff training are essential to mitigate these risks and ensure the security of patient data in an AI-driven healthcare environment. Healthcare organizations must also have well-defined incident response plans to address breaches promptly and minimize their impact [30].

Informed consent: The use of AI in healthcare requires patients to provide informed consent, indicating that they understand the implications of AI-driven care. However, ensuring that patients fully comprehend the intricacies of AI can be challenging. AI systems often involve complex algorithms and large datasets that may need to be explained to patients. To address this, healthcare providers and organizations must invest in patient education and transparent communication. They should inform patients about the role of AI in their care, its potential benefits, and the safeguards in place to protect their data. Providing patients with clear and accessible information is essential to obtaining informed consent and respecting patient autonomy while incorporating AI into healthcare practices. Patients must have confidence in AI systems and trust that their data will be handled securely and ethically [31].

Ethical Considerations in AI-Driven Decision-Making

Transparency: The transparency of AI algorithms is a critical ethical consideration in healthcare. AI systems can be highly complex, making it challenging to explain their decisions to healthcare professionals and patients. Transparency is essential to gain trust and confidence in AI-driven healthcare. Patients have the right to understand why an AI system recommends a particular treatment or diagnosis. Healthcare professionals also need to comprehend AI decisions to make informed clinical judgments. Providing clear explanations of AI processes and decisions, as well as disclosing any limitations, is vital to ensure that AI in healthcare is both practical and trusted [32].

Accountability: Determining accountability in AI-driven healthcare is a complex ethical issue that needs clarification. When AI systems make decisions that impact patient care, it is essential to establish who is responsible for these decisions and any associated errors or biases. Healthcare professionals, developers of AI systems, and healthcare organizations all play a role in AI accountability. Developing clear guidelines for the roles and responsibilities of each party is crucial to ensure that the right individuals or entities are held accountable for AI-related outcomes. Accountability frameworks can help address concerns related to legal, ethical, and medical liability [33].

Fairness: Ensuring that AI systems do not perpetuate or exacerbate healthcare disparities or biases is a critical ethical concern. AI must be designed and trained to provide equitable care to all patients, regardless of race, gender, socioeconomic status, or other demographic factors. Ethical considerations include addressing issues such as algorithmic bias and healthcare disparities. AI developers and healthcare organizations must actively work to reduce biases in AI systems and continuously monitor and audit their performance to ensure fairness. Ethical guidelines should be in place to promote equitable healthcare delivery and to mitigate the risk of underserved or disadvantaged populations being further marginalized by AI-driven healthcare [34].

Bias and discrimination: AI algorithms can potentially inherit biases from the data they are trained on. These biases can lead to unfair or discriminatory decisions in healthcare, affecting diagnosis, treatment, and patient care. Ethical considerations include actively addressing and mitigating these biases. Healthcare organizations should carefully curate training data to minimize bias, and AI developers should incorporate bias detection and mitigation mechanisms into their systems. Regular audits and testing for bias in AI algorithms are essential to ensure that healthcare decisions are just, fair, and free from discrimination. AI systems must be held to high ethical standards to prevent bias and discrimination in healthcare [35].

Challenges Related to AI Adoption in Healthcare Institutions

Resource constraints: Resource constraints are a significant challenge, especially for smaller healthcare institutions. The implementation of AI systems often requires a substantial financial investment in both technology and staff training. Smaller healthcare facilities may need help to allocate the necessary budget and human resources to adopt and maintain AI solutions. Overcoming this challenge may involve seeking external funding partnerships or exploring cost-effective AI solutions tailored to the specific needs and constraints of smaller healthcare organizations [36].

Integration with existing systems: Adapting AI technologies to work seamlessly with existing healthcare IT systems and workflows is complex and time-consuming. Healthcare institutions often have legacy systems, and integrating AI solutions can disrupt existing operations. Achieving a smooth integration that minimizes disruptions while maximizing the benefits of AI is a significant challenge. Healthcare organizations must invest in interoperable systems and consider gradual implementation to facilitate a smoother transition [37].

Resistance to change: Resistance to change is a common challenge in adopting AI in healthcare. Healthcare professionals may have concerns about job security, fearing that AI could replace some aspects of their work. There may also be skepticism about the effectiveness of AI technologies in improving patient care. Addressing these concerns requires comprehensive education and training programs to ensure that healthcare professionals understand the role of AI as a tool to enhance their work rather than replace it. Engaging healthcare workers in the AI adoption process, listening to their feedback, and involving them in AI system design and implementation can help mitigate resistance to change [38].

Regulatory compliance: The healthcare sector is heavily regulated, and navigating the complex regulatory environment, such as HIPAA in the United States, is a significant challenge when implementing AI solutions. Ensuring that AI implementations comply with legal requirements, protect patient privacy, and maintain data security is essential but can be demanding. Healthcare organizations must work closely with legal and compliance experts to ensure AI solutions align with regulations. This may involve conducting thorough risk assessments, data security audits, and ongoing monitoring to maintain compliance while benefiting from AI technologies [39].

Addressing Biases and Disparities in AI Algorithms and Datasets

Data bias: Data bias is a significant challenge in AI-driven healthcare. AI algorithms rely on training data, and if this data is biased or unrepresentative of diverse populations, it can lead to disparities in healthcare decisions. Addressing this challenge requires a concerted effort to ensure that training datasets are diverse and representative. Healthcare organizations should actively curate data to minimize bias, incorporating a wide range of demographic and clinical information to create more inclusive and equitable AI models. Ongoing data quality assessments and monitoring are essential to identify and rectify bias in training data [40].

Algorithmic bias mitigation: Developing techniques to mitigate algorithmic bias and prevent AI systems from making biased decisions is an ongoing ethical challenge. AI developers must work to identify and address bias at multiple stages of the AI development process, from data collection and preprocessing to algorithm design and model evaluation. This includes considering factors contributing to bias, such as underrepresented groups and historical disparities in healthcare. AI models should be designed to minimize bias and promote fairness in decision-making, which may involve using specialized bias mitigation techniques and algorithms [41].

Fairness auditing: Implementing methods to audit AI systems for fairness and bias on an ongoing basis is essential to maintain equitable healthcare outcomes. Fairness auditing involves continuously monitoring AI models for bias and disparities in decisions and outcomes. Audits can help identify and rectify issues as they arise, ensuring that AI systems remain fair and just. This process may require the development of specialized fairness metrics, transparency in AI decision-making, and routine assessments by ethics committees or regulatory bodies to validate fairness in healthcare AI [42].

Ethical guidelines: Establishing and adhering to ethical guidelines for AI development and use is a foundational step in mitigating bias and disparities. These guidelines can provide a framework for AI developers, healthcare organizations, and regulatory bodies to ensure that AI systems prioritize fairness, equity, and patient well-being. Ethical guidelines should address data collection, algorithm design, model evaluation, and the rights and responsibilities of healthcare professionals and patients. Adherence to these guidelines can be a foundational strategy for reducing bias and disparities in AI-driven healthcare and promoting ethical and equitable AI practices [43].

Future prospects and opportunities for AI in nursing science and healthcare

Potential Advancements and Innovations in AI Technologies

AI-driven diagnostics: Advances in AI algorithms and machine learning are poised to revolutionize diagnostics in healthcare. These technologies can potentially deliver even more accurate and faster diagnostic tools, enhancing the early detection of diseases. AI can analyze vast datasets, including medical images, patient records, and genetic information, to identify subtle patterns and indicators that human practitioners might miss. Improved diagnostic accuracy can lead to earlier interventions and more successful treatment outcomes, ultimately improving patient health and reducing the burden on the healthcare system [2].

AI-enhanced robotic surgery: Innovations in surgical robotics and AI guidance systems can potentially transform the field of surgery. AI can provide real-time guidance to surgeons, enhancing precision and enabling minimally invasive procedures. This not only reduces recovery times but also lowers the risk of complications and post-operative pain for patients. AI-enhanced robotic surgery represents a significant advancement in healthcare, offering patients safer and more effective treatment options while improving the quality of care provided by healthcare professionals [44].

Predictive analytics: Future AI models will likely offer more precise and real-time predictions for patient outcomes. By continuously monitoring patient data, including vital signs, medical history, and environmental factors, AI can predict health events, such as disease exacerbations or hospital admissions. These predictive analytics can empower healthcare providers to adopt proactive and preventive care strategies, intervening before conditions worsen. Patients benefit from improved health management, reduced hospitalizations, and better quality of life, while healthcare organizations can optimize resource allocation and patient care [2].

Quantum computing: The emergence of quantum computing holds immense potential for accelerating AI-driven healthcare research and simulations. Quantum computing's unprecedented processing power can handle complex AI models and large healthcare datasets more efficiently. This can lead to groundbreaking discoveries in drug development, disease modeling, and treatment optimization. Quantum computing can significantly shorten the time required for medical research and enhance the precision of AI algorithms. This technological synergy may bring us closer to innovative healthcare solutions and more effective treatments for various conditions [45].

Integration of AI With Emerging Healthcare Practices and Technologies

Telemedicine and remote care: AI will continue to enhance telemedicine and remote care. This technology allows for more sophisticated remote consultations, diagnostics, and monitoring. AI-powered virtual healthcare assistants can support healthcare providers by triaging patients, offering preliminary diagnoses, and providing real-time data analysis during telehealth visits. Patients can access more comprehensive and accurate care without physical visits to healthcare facilities. This not only improves patient access to care but also helps manage the demand for healthcare services, especially in cases where in-person visits may be challenging or unnecessary [46].

IoT and wearable devices: Integrating AI with the Internet of Things (IoT) and wearable devices can provide more comprehensive and real-time health monitoring. AI can analyze the data collected by these devices, such as heart rate, blood pressure, and activity levels, to provide valuable insights into a patient's health. This benefits patients and healthcare providers by enabling early detection of health issues, tracking chronic conditions, and offering personalized health recommendations. Healthcare professionals can use this data to make more informed decisions, and patients gain a better understanding of their health, leading to improved overall healthcare outcomes [47].

Blockchain and data security: AI can be employed in ensuring the security of patient data in blockchain-based healthcare systems. By using AI to monitor and protect the integrity of healthcare data stored in distributed ledgers, the trust and data security of these systems can be enhanced. Blockchain technology offers a secure and transparent method for storing and sharing healthcare data, and AI can further strengthen data security by detecting and preventing unauthorized access or alterations. This combination of technologies promotes data integrity, patient privacy, and secure healthcare data sharing, vital aspects of modern healthcare systems [48].

Genomic medicine: AI is expected to play an even more significant role in advancing the analysis of genomic data. Genomic medicine, which focuses on tailoring healthcare to an individual's genetic makeup, can be significantly facilitated by AI. AI-driven genomic analysis allows for identifying genetic predispositions, disease markers, and treatment options personalized to an individual's unique genetic profile. This approach is particularly promising in cancer treatment, rare disease diagnosis, and drug development. AI can expedite the analysis of vast genomic datasets and enable healthcare providers to make more precise and personalized treatment decisions, improving patient outcomes and revolutionizing the field of genomics [49].

Implications for the Future of Nursing Education and Training

AI-integrated curricula: Nursing education must evolve to incorporate AI-related coursework into curricula. This is essential to ensure that future nurses are proficient in using AI technologies effectively and ethically. Courses in AI fundamentals, data analysis, and AI applications in healthcare can prepare nursing students to work with AI-driven tools and systems. Ethical considerations, such as patient data privacy and bias mitigation, should also be integral to the curriculum. AI-integrated education equips nurses with the skills and knowledge to navigate the evolving healthcare landscape and provide high-quality, AI-augmented care [50].

Continuing education: Ongoing training and upskilling opportunities will be crucial for nurses to stay updated on the latest AI applications and healthcare technologies. The rapid pace of AI development and its integration into healthcare necessitates continuous learning. Healthcare organizations should provide nurses with AI training and professional development opportunities. This ensures that nurses remain current in their knowledge and can adapt to new AI tools and practices. Continuing education not only benefits nurses but also improves patient care by enabling healthcare professionals to leverage the latest AI advancements [51].

AI-assisted learning: AI-powered educational tools may become an integral part of nursing education. These tools can offer personalized learning experiences and support for nursing students. AI can adapt educational content to individual learning styles and progress, offering targeted guidance and resources. This enhances the effectiveness of nursing education and helps students gain a deeper understanding of AI's role in healthcare. AI-assisted learning not only improves the quality of education but also prepares nurses to work alongside AI systems in clinical practice [52].

Interdisciplinary training: Collaborative training involving nurses, data scientists, and AI experts may become familiar to ensure effective teamwork in healthcare settings. Nursing professionals increasingly work in multidisciplinary healthcare teams, collaborating with data scientists, AI developers, and other experts. Interdisciplinary training programs can help nurses understand the capabilities and limitations of AI, foster effective communication across different specialties, and encourage collaboration to achieve the best patient outcomes. Such training promotes a holistic approach to healthcare and ensures that AI is integrated seamlessly into the healthcare ecosystem [53].

Collaborative Approaches to Harnessing AI's Full Potential in Healthcare

Interdisciplinary research: Collaborations between healthcare professionals, data scientists, and engineers will play a pivotal role in driving AI's role in healthcare. Interdisciplinary research teams bring together diverse expertise to address complex healthcare challenges. Healthcare professionals provide domain knowledge, data scientists offer AI and data analysis skills, and engineers develop AI-powered technologies. Together, they can innovate and develop solutions that enhance patient care, improve diagnostics, and optimize healthcare workflows. These collaborations will foster the development of AI systems that are not only effective but also align with the practical needs of healthcare [54].

Regulatory frameworks: Policymakers, healthcare institutions, and technology developers must work together to create and enforce ethical and regulatory frameworks for AI in healthcare. These frameworks provide the guidance and rules necessary to ensure the responsible and secure use of AI in healthcare settings. Ethical considerations, data privacy, and transparency must be at the forefront of regulatory discussions. By establishing clear guidelines and standards, stakeholders can foster trust in AI-driven healthcare and ensure that AI technologies are deployed in a way that prioritizes patient safety, privacy, and equitable care [43].

Patient involvement: Engaging patients in discussions about AI's role in their healthcare is vital for achieving successful AI implementation. Patients should have a voice in decisions related to the use of AI in their care. This includes understanding how AI technologies work, obtaining informed consent for AI-driven procedures, and respecting patients' preferences regarding the involvement of AI in their healthcare. Patient-centered AI applications can lead to higher satisfaction, improved communication between patients and healthcare providers, and more effective healthcare delivery [55].

Knowledge sharing: Open sharing of AI research, data, and models can accelerate progress and improve the overall quality of AI applications in healthcare. Collaboration and knowledge sharing among researchers, healthcare organizations, and AI developers foster innovation and help avoid duplication of efforts. Open-access datasets, open-source AI models, and collaborative research efforts can collectively advance the field of healthcare AI. This not only accelerates the development of AI solutions but also enables researchers and practitioners to learn from each other's experiences, leading to the development of more effective and ethical AI systems in healthcare [56].

Recommendations for optimizing AI integration in nursing science and healthcare

Strategies for Addressing Challenges and Ethical Concerns

Transparent governance: Establishing clear governance structures within healthcare organizations is essential to oversee AI implementations. These structures ensure transparency, accountability, and ethical decision-making using AI technologies. Governing bodies can define guidelines, ethical principles, and policies for AI applications in healthcare. These structures also play a crucial role in ensuring that AI systems align with the organization's mission and values while focusing on patient safety, data security, and ethical patient care [57].

Regular auditing: Routine audits of AI systems are vital to identify and rectify biases, disparities, and potential ethical concerns in decision-making processes. These audits involve monitoring AI algorithms for any signs of bias, discrimination, or unintended consequences. Regular assessments help maintain AI system fairness, integrity, and adherence to ethical standards. Dedicated teams or interdisciplinary committees should perform auditing to ensure that AI systems continue to provide equitable care to all patients [58].

Ethics committees: Forming interdisciplinary ethics committees is a valuable approach to review and guide AI applications in healthcare. These committees typically include healthcare professionals, AI experts, ethicists, and other relevant stakeholders. They play a crucial role in evaluating the ethical implications of AI in healthcare, assessing AI algorithms and models, and offering guidance on ethical decisions related to patient care. Ethics committees can provide invaluable insights into complex ethical dilemmas, ensuring that AI systems in healthcare align with the highest ethical standards and patient well-being [59].

Bias mitigation: Implementing strategies for identifying and mitigating biases in AI algorithms is crucial, with a focus on diversity and fairness. Bias mitigation involves regular data quality checks, algorithmic audits, and the development of strategies to reduce both evident and subtle biases in AI decision-making. Techniques such as fairness-aware machine learning can help AI developers create models that prioritize equitable patient care. Mitigating bias is a continuous process, and healthcare organizations must work to ensure that AI systems remain fair and just, offering equal opportunities and care to all patients [60].

Training and Education Initiatives for Healthcare Professionals

AI training: Integrating AI-related training into nursing and medical curricula is essential to prepare healthcare professionals to work effectively with AI technologies. Healthcare education programs should include courses on AI fundamentals, data analysis, and the practical applications of AI in healthcare. This training equips future nurses and physicians with the knowledge and skills needed to navigate AI-driven healthcare, ensuring that they can leverage these technologies to enhance patient care. Additionally, addressing ethical considerations and issues related to data privacy and bias is crucial to preparing healthcare professionals to use AI responsibly [61].

Continuing education: Offering ongoing education and training opportunities is vital for healthcare professionals to stay current with AI advancements and ethical considerations. The field of AI is rapidly evolving, and healthcare professionals must continuously update their knowledge and skills to utilize the latest AI tools and practices effectively. Continuous learning programs can provide healthcare professionals access to the most up-to-date information on AI applications, ensuring that they remain at the forefront of patient care and can adapt to emerging technologies [2].

Interdisciplinary workshops: Promoting interdisciplinary workshops and collaboration between healthcare professionals and AI experts fosters a deep understanding of AI's capabilities and limitations. These workshops enable healthcare practitioners to work closely with data scientists, AI engineers, and researchers. Collaborative efforts provide insights into how AI technologies work, the challenges they pose, and the opportunities they offer. Interdisciplinary workshops enhance the ability of healthcare professionals to communicate with AI experts effectively and contribute to the development and evaluation of AI systems in healthcare settings [62].

Patient-centered communication: Training healthcare professionals in effectively communicating AI-driven care decisions to patients is essential. Healthcare providers must be able to explain the role of AI in patient care, address any concerns or questions, and obtain informed consent when AI is involved in diagnosis or treatment. Patient-centered communication is critical to ensure patients understand and are comfortable using AI in their healthcare. It fosters trust and transparency, allowing patients to participate actively in their care decisions, ultimately leading to more positive healthcare experiences. This training empowers healthcare professionals to engage in open and informed discussions with patients regarding AI-related care [63].

Guidelines for Responsible AI Implementation in Healthcare Settings

Data privacy: To safeguard patient information in AI-driven healthcare systems, organizations should develop and enforce strict data privacy and security protocols. These protocols should encompass encryption, access controls, secure storage, and data transmission. Compliance with regulations such as the Health Insurance Portability and Accountability Act (HIPAA) in the United States is crucial. Implementing robust data privacy measures ensures patient data remains confidential and secure, building trust in AI-driven healthcare systems [64].

Algorithm transparency: Guidelines for ensuring transparency in AI algorithms are essential to enable healthcare professionals and patients to understand the reasoning behind AI-driven recommendations. Healthcare organizations should prioritize the development of AI models that are interpretable and explainable. This transparency allows healthcare professionals to trust and effectively use AI systems while patients can be informed and involved in care decisions. Algorithm transparency supports ethical and accountable AI-driven healthcare [65].

Fairness and bias guidelines: Healthcare organizations should establish guidelines for identifying and addressing bias in AI algorithms. Bias can lead to disparities in healthcare decisions, which is unacceptable. These guidelines should include practices for fair data collection, model evaluation, and ongoing bias mitigation. Developing AI systems prioritizing fairness and equity in care ensures patients receive treatment free from bias and discrimination [35].

Standardized data collection: Encouraging standardized data collection and reporting practices is essential to enhance the quality and consistency of healthcare data used by AI systems. Standardization involves defining uniform data formats, terminologies, and protocols for data collection. This ensures that data are interoperable and reliable, facilitating the training and performance of AI algorithms. Standardized data collection not only benefits AI-driven healthcare but also improves data sharing and collaboration among healthcare institutions, ultimately leading to better patient care and research outcomes [66].

Policy Recommendations for Promoting AI Adoption and Regulation

Regulatory frameworks: Developing comprehensive and adaptable regulatory frameworks is essential to address AI in healthcare. These frameworks should encompass guidelines for testing, validating, and approving AI applications in healthcare. Such regulations should define the requirements for AI safety, efficacy, data privacy, and ethical considerations. Flexibility is crucial to accommodate the rapid pace of AI innovation, allowing regulations to adapt to evolving technologies while ensuring patient safety and data security [67].

Incentives for innovation: To promote the development of AI technologies for healthcare, governments and healthcare organizations should offer incentives. These incentives can include research grants, tax credits, and funding opportunities for AI-focused research and development in healthcare. By providing financial support for innovation, stakeholders encourage AI experts and organizations to invest in developing healthcare-related AI solutions, leading to improved patient care, more efficient healthcare systems, and economic benefits [68].

Patient rights: Advocating for policies that protect patients' rights is crucial in AI-driven healthcare. Patients should have the right to access, control, and have a say in how their data is used in AI-driven healthcare. This includes the ability to grant or withdraw consent for AI-driven procedures, access their healthcare data, and know how their data is being utilized. Patient rights policies should ensure transparency, informed consent, and data portability, empowering patients to make informed choices about their healthcare and data privacy [64].

Cross-border collaboration: Promoting international collaboration on AI regulation is vital to facilitate the sharing of best practices and ensure global standards in healthcare. AI knows no borders, and global cooperation is essential to harmonious regulations and standards across different countries. Collaborative efforts enable the exchange of knowledge and experiences, fostering the development of global guidelines for the ethical and responsible use of AI in healthcare. This not only benefits healthcare professionals but also ensures consistent and high-quality patient care on a global scale [69].

## Conclusions

In conclusion, our comprehensive review of AI's impact and challenges in nursing science and healthcare has unveiled a landscape brimming with potential and complexity. AI's capacity to improve patient outcomes, enhance efficiency, and propel medical research is undeniable. However, the ethical considerations surrounding transparency, fairness, and accountability remain at the forefront of discussions. Addressing data privacy, AI adoption challenges and bias mitigation is imperative to optimize AI's integration in healthcare settings. The future of nursing science and healthcare holds exciting prospects, including AI-driven diagnostics and predictive analytics, which could revolutionize patient care. Nevertheless, it is vital to underscore that AI complements human healthcare providers, not a substitute, as the human touch and empathy remain irreplaceable. The call to action is clear: continued research, interdisciplinary collaboration, ethical guidelines, and the protection of patient rights are essential to realize AI's full potential in healthcare and drive improvements in patient outcomes, accessibility, and quality of care.
